# P-497. Iron Supplementation in Pediatric HIV: a Systematic Review and Meta-Analysis Balancing Nutritional Benefits and Infection Progression

**DOI:** 10.1093/ofid/ofaf695.712

**Published:** 2026-01-11

**Authors:** Leticia R Campos, Gisella Carpi, Sophia Costa, Thiago Netto, Jose Luis Boene, Oscar Hernández Rios, Taniela M Bes

**Affiliations:** Universidade de Ribeirão Preto (UNAERP), Ribeirão Preto, Sao Paulo, Brazil; Hospital de Clínicas de Porto Alegre (HCPA), Porto Alegre, Rio Grande do Sul, Brazil; Jose Lucas Municipal Hospital, Belo Horizonte, Minas Gerais, Brazil; Evandro Chagas National Institute of Infectious Diseases (Fiocruz), Rio de Janeiro, Rio de Janeiro, Brazil; Eduardo Mondlane University, Maputo, Maputo, Mozambique; Ricardo Palma University, Lima, Lima, Peru; MetroWest Medical Center, Framighan, MA

## Abstract

**Background:**

Children living with HIV face a dual clinical challenge: they require iron to support neurodevelopment and prevent anemia, yet excess iron may promote viral replication through oxidative stress and immune modulation. This systematic review and meta-analysis (MA) aimed to evaluate the effects of iron supplementation on immunological markers and anemia outcomes in HIV-infected pediatric populations, focusing on CD4 counts and hemoglobin (Hb) correction.

**Figure:**
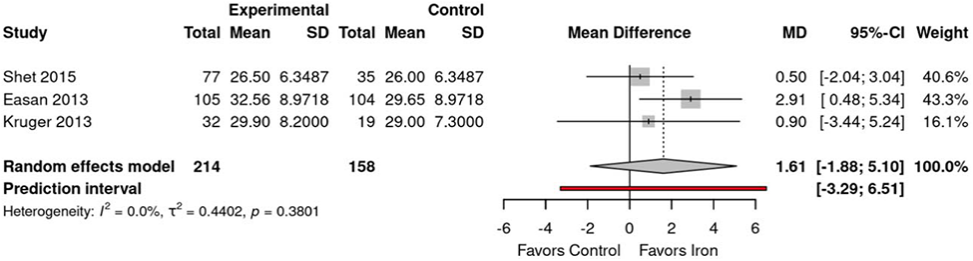
Forest Plot comparing CD4 count in experimental group x control group Iron supplementation in HIV-positive children does not adversely affecting CD4 counts.

**Figure:**
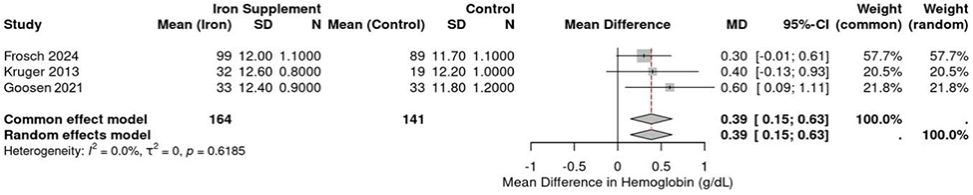
Forest Plot comparing hemoglobin levels in experimental group x control group Iron supplementation in HIV-positive children improves hemoglobin levels

**Methods:**

A systematic search of indexed databases identified clinical studies evaluating iron supplementation in children with HIV. Of 204 screened records, six studies met the inclusion criteria. Primary outcomes assessed CD4 counts and percentages, with additional analysis focused on Hb levels. The MA was conducted using a random-effects model in R software, calculating standard mean differences with 95% confidence intervals. The Hartung-Knapp adjustment was applied for conservative confidence intervals, while sensitivity analyses assessed the impact of estimated standard deviations on the pooled effect estimates.

**Results:**

MA revealed divergent outcomes across endpoints. For CD4 percentage, iron supplementation showed no significant effect, with a pooled mean difference of +1.61% (*95% CI: –1.88 to 5.10*, *p* = 0.38), and no statistical heterogeneity (*I² = 0.0%*, *τ² = 0.4402*, *p* = 0.38). Only Esan (2013) demonstrated a statistically significant CD4 improvement (*MD: +2.91%*, *95% CI: 0.48 to 5.34*, *p* = 0.02), limited to the first 3 months. In contrast, iron supplementation significantly increased Hb concentration by 0.39 g/dL (*95% CI: 0.15 to 0.63*), consistently favoring iron across all included studies. No heterogeneity was observed (*I² = 0%*, *p* = 0.62), reinforcing the strength and consistency of this effect.

**Conclusion:**

Iron supplementation appears to be an effective strategy to correct anemia in children living with HIV, without evidence of harm to immunologic status based on current data. However, given the small number of studies and mixed findings regarding CD4 response, clinicians should apply iron therapy with careful monitoring. Further large-scale, long-term studies are essential to determine whether these short-term hematologic benefits translate into improved clinical outcomes and immunologic safety.

**Disclosures:**

All Authors: No reported disclosures

